# Application of an arched, Ni–Ti shape-memory connector in repairing distal tibiofibular syndesmosis ligament injury

**DOI:** 10.1186/s12891-022-05449-9

**Published:** 2022-05-19

**Authors:** Jinbo Zhao, Yuntong Zhang, Yan Xia, Xuhui Wang, Shuogui Xu, Yang Xie

**Affiliations:** Department of Orthopedics, The First Affiliated Hospital of Naval Military Medical University, Shanghai, 20000 China

**Keywords:** Ni–Ti Arched shape-memory Connector, Distal tibiofibular syndesmosis ligament, Ankle, Internal fixation

## Abstract

**Objective:**

To investigate the clinical effect of internal fixation of a Ni–Ti arched shape-memory connector in the treatment of distal tibiofibular syndesmosis ligament injury.

**Methods:**

From January 2013 to January 2016, 108 cases of ankle fracture with distal tibiofibular syndesmosis ligament injury in our hospital were selected, and all of them were fixed with ASCs or screw fixation. The functional evaluation and efficacy evaluation were performed according to the Olerud Molander Ankle Score (Omas) and SF-36. At the same time, follow-ups recorded the incidence of postoperative complications: osteoarthritis, superficial infection, symptomatic hard and soft tissue irritation, early removal and poor reduction of internal fixation, and later loss of reduction.

**Results:**

In the ASC(Ni–Ti Arched shape-memory Connector) group, the incidence of symptomatic hardware, soft tissue or superficial infection decreased to 2.77%(from 13.8% or 11.1% in SCREW group). The early removal rate(2.77%) of internal fixation was also lower than that of the screw group. While the incidence of osteoarthritis is 13.8% in SCREW group, the incidence of osteoarthritis in the later follow-up was also as low as 1.38% in ASC group. Loss of fracture reduction due to removal of the fixation device for the distal tibiofibular syndesmosis ligament was not observed in the ASC group. With two postoperative scoring systems (OMAS and SF-36), patients in the ASC group significantly get higher score than that in SCREW group.

**Conclusion:**

The design of the Ni–Ti arched shape-memory connector can be adapted to the irregular anatomical structure of the malleolus and the ability to continue to contract by body temperature. The use of ASCs in fixation of articular ligaments can preserve a slight range of motion, and the results suggest that ASCs can effectively reduce the incidence of fixation looseness, fracture, infection and other complications.

## Introduction

Ankle injuries usually involve ligaments related to the tibia and fibula, of which the joint ligament of the inferior tibia and fibula is the more difficult to detect. These ligaments are named the anterior inferior tibiofibular syndesmosis ligament(AITFL), posterior-distal tibiofibular syndesmosis ligament, inferior transverse tibiofibular syndesmosis ligament and interosseous ligament. These ligaments ensure stability and relative movement between the tibia and fibula. This group of ligaments also helps the tibia and fibula sustain the weight of the body and are key ligaments in the ankle joint [1].

The distal tibifibular ligament is strained during external pronation and hyperdorsiflexion of the ankle and is therefore often associated with pronation and supination ankle fractures [2, 3]. Syndesmosis injuries occur in 10% of ankle fractures and in approximately 20% of ankle fractures requiring internal fixation. Anterior tibiofibular syndesmosis ligament and interosseous membranes will fracture if ankle injury is serious enough [4]. Severe ligament injuries and tears can lead to subluxation of the distal tibiofibular joint, widening of the ankle mortise, and loss of normal correspondence of the tibiotalar joint. Without reduction and proper fixation, ankle instability, residual pain, and an increased incidence of traumatic arthritis can occur.

In summary, a robust and reliable internal fixation device for unstable ankle fractures is effective in reducing traumatic osteoarthritis and improving later functional outcomes [5]. Currently, there are a variety of fixation devices for the distal tibiofibular joint, and the most widely used is the distal tibiofibular screw attached to the lateral fibular plate. However, fracture of the tibiofibular screw is frequently reported in the literature [6] and forced routine removal of the screw causes wound infection and other complications in approximately 9.2% of patients [7]. Only some of the literature supports early and complete removal of distal tibiofibular fixation as being able to reduce the incidence of complications [8]. Another form of treatment is the suture button system, also known as tightrope, which repairs the tibiofibular joint while giving the joints proper mobility. Buttons, however, are located below the surface of the skin and have been reported to have a higher incidence of skin irritation and infection. Loosening of the fixation cord between the buttons, causing the joint fixation device to lose its fixation effect, is also a common complication [9]. In addition to the above two internal fixation devices, syndesmosis elastic hook and absorbable micro internal fixation methods are used for the treatment of inferior tibiofibular syndesmosis ligament injury.

Over the past several decades, arched Ni-Ti shape-memory connectors have been widely fabricated and applied in various clinical fields, including orthopaedic, stomatological, urological, and cardiovascular fields, due to their excellent properties of wear and corrosion resistance, good biocompatibility, and shape memory effects [10–17].Therefore, by learning from the Ni-Ti arched shape-memory connector (ASC) used in other fractures [18, 19], we attempted to fix the position of the distal tibiofibular joint using ASCs to repair syndesmotic ligament. The purpose of our research group is to investigate the clinical effect of internal fixation of a Ni-Ti arched shape-memory connector in the treatment of distal tibiofibular syndesmosis ligament injury.

## Materials and methods

Retrospective analysis was conducted on patients with ankle fracture complicated with distal tibiofibular syndesmosis ligament injury in our hospital from 2013 to 2016. Through X-ray and CT scans, compression tests, external rotation stress tests, and intraoperative Cotton tests, the patient was diagnosed with a fracture of the ankle joint with an injury to the inferior tibiofibular syndesmosis ligament. Such patients were collected and analysed, and the sample cases that did not conform to the retrospective study were deleted according to the following exclusion criteria: ①pathological osteoporosis; ②severe infection; ③receiving long-term hormone therapy. After clean-up, 108 patients were included. There were 59 males and 49 females, aged 20-76 years old. Causes of injury included 53 cases of traffic accidents, 36 cases of sports sprain and 9 cases of fall injuries. Other detailed clinical data are listed in Table 1. A total of 108 patients were divided into screw or ASC groups according to different surgical methods (72 patients in SCREW group while 36 patients in ASC group). According to the Lauge-Hansen classification, among the 108 patients in the whole group, there were 62 cases of pronation and external rotation classification, and 46 cases of suppination external rotation classification. The mean follow-up time from surgery was 42 ± 11 months.

**Table 1:** Clinical basis and prognosis-related data analysis

Total number	ASC	Screw	*P* value
	72	36	
Age			
< 65	41	22	0.836
> = 65	31	14	
Gender			
Male	36	23	0.245
Female	36	13	
BMI	22.78 ± 3.18	22.15 ± 3.33	0.334
Lauge-hansen type			
Pronation and external rotation	44	18	0.371
Suppination external rotation	28	18	
Osteoarthritis	1(1.38%)	5(13.8%)	
Early removal	2(2.77%)	36(100%)	
Symptomatic hardware and Soft tissue irritation	2(2.77%)	5(13.8%)	
Superficial infection	2(2.77%)	4(11.1%)	
Malreduction	1(1.38%)	5(13.8%)	
Loss of reduction	0	4(11.1%)	

Due to the different types of data, we use different statistical methods. For the enumeration data, we used chi-square statistics, and for the measurement data obtained by the scoring system, we used T-test to evaluate the postoperative prognosis of the two groups. The *P* value obtained only reflects the statistical difference between the two groups. *P* value less than 0.05 was considered to be statistically different between the two groups.

Tip:X-ray diagnostic criteria: 1. The gap between the upper and lower tibia and fibula is less than or equal to 6 cm on the anterior and posterior or ankle acupoint films; 2. The overlap of the tibia and fibula on the anterior and posterior views is greater than 6 cm or greater than 42% of the width of the fibula; 3. The overlapping shadow of the tibia and fibula on the ankle acupoint film is greater than 1 cm; If the above range is exceeded, the lower tibia and fibula are separated.

All methods were carried out in accordance with regulations or declaration of Helsinki.

### Surgical procedure

**Fig. 1:**
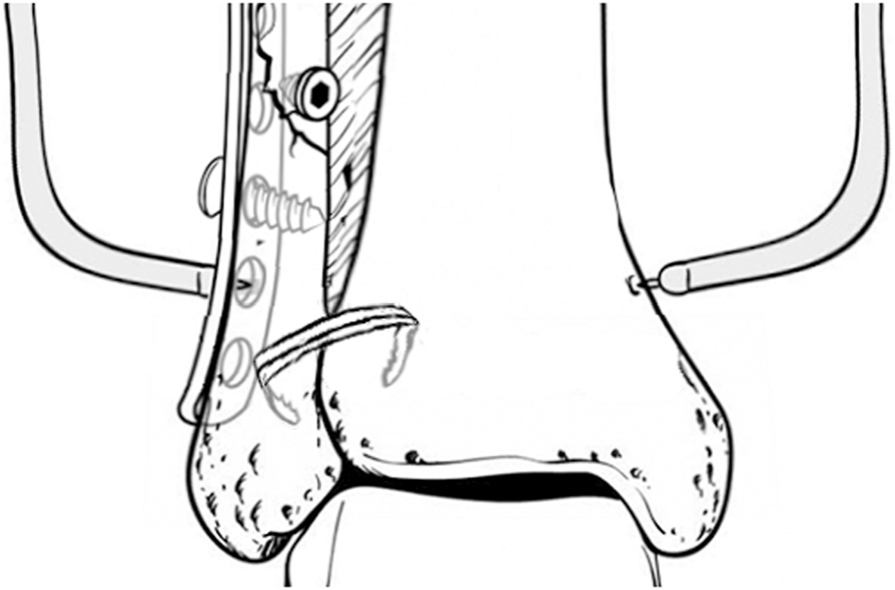
ASCs were used to reduce and fix the distal tibiofibular ligament between the tibiofibular bones

A total of 108 cases of ankle joint fractures and distal tibifibular syndesmosis ligament injuries underwent open reduction and internal fixation under the guidance of two professors in our department. The difference is that the screw group implemented traditional screw fixation of the distal tibifibular joint, while the ASC group implemented ASC fixation. Here, we describe in detail the reduction and fixation of the distal tibiofibular syndesmosis ligament in patients with ASC. After standard methods of reduction of the fibular and tibial fractures were performed, the distal tibiofibular joint was reduced under direct visualization of the syndesmosis and held at its anatomical position by periarticular reduction forceps. The ankle joint was positioned at an angle of 90° between the tibial shaft and the foot during syndesmosis reduction and fixation. Two holes with diameters of 1.5 to 2.0 mm (According to the size of the ASC spike teeth) were drilled bilaterally on the fibula and tibia. Then, the Ni-Ti arched shape-memory connector (ASC) was placed in cold (0–4 °C) water to allow plastic deformation of the Ni-Ti alloy. The claws were unfolded. Appropriate sized ASCs were implanted in the holes. Warm water (40 °C) was applied for irrigation and compression. The memory alloy creates a continuous fixation force triggered by body temperature. Then, a fluoroscopic stress test was used to confirm the syndesmotic reduction. (Fig. 1)

Postoperatively, the affected limb was elevated for 3 days. For most patients, gradual full-range mobilization without weight bearing was recommended as soon as possible under the supervision of a qualified physiotherapist, and patients were encouraged to perform progressive active exercises. Partial or full weight bearing was allowed between three and four weeks after surgery, depending on patient tolerance and fracture stability. Follow-up was obtained at 1, 3, 6, and 12 months after surgery and yearly thereafter. Plain anteroposterior (AP) and lateral radiographs were obtained at each visit. All changes in the position of the implant and reduction, fixation failures and removal or complications were recorded. At the latest follow-up, functional outcomes were assessed by one of the authors using the Olerud Molander Ankle Score (OMAS) [20, 21]. The Medical Outcomes Study 36-item short-form health survey (MOS SF-36) questionnaire was chosen to assess the effect of ankle fractures and lesions on health-related quality of life at the same time [22].

## Results

No patients were lost to follow-up. The mean follow-up time from surgery was 42 ± 11 months. SPSS software was used to analyse the basic clinical information of patients in the two groups, and we confirmed that there was no statistically significant difference in the basic clinical information, such as sex, age, BMI and other indicators, between the two groups.

During our follow-up, we had only one case of osteoarthritis in the ASC group, while in the screw group, we found 5 (13.8%) patients with delayed osteoarthritis, and the data were statistically significant.In addition, the screws need to be routinely removed 1-2 months after surgery. The device removal rate was 100%, while in the ASC group, the early removal rate was only 2.77% (2 cases), which greatly reduced the number of operations and costs for patients. The application of ASC was small and located on the surface of the bone, so there was no need to worry about the problem that the fractured ASC cannot be removed. At the same time, in the follow-up of complications in the ASC group, we found that the incidence of symptomatic hardware and soft tissue irritation was only 2.77% and the occurrence of superficial infection was the same, and the incidence of related complications in the SCREW group was 13.8% and 11.1%, respectively. We performed AP assessment for the joint reduction of patients more than one month after the operation and half a year later. We found that the probability of poor reduction and loss of reduction in the screw group was 13.8% and 11.1%, respectively, while in the ASC group, we found only one patient with symptoms of loss of reduction. Statistical analysis of the data are included in Table 1.

To better understand the patient's prognosis and experience, we used the SF-36 and scoring rules for OMAS statistical prognosis. In OMAS score system, patients in ASC group significantly get higher scores than SCREW group. And also, scores collected in ASC group by MOS SF-36 scoring system performs better than that of SCREW group. (Table 2)

**Table 2:** Data analysis of OMAS and SF-36 scores

Parameter	Operation method	number of cases	Average	Standard Deviation	*P* value
OMAS	ASC	72	85.51	6.606	0.012
	Screw	36	81.97	7.229	
SF-36	ASC	72	85.21	6.068	0.000
	Screw	36	79.72	5.273	

**Fig. 2:**
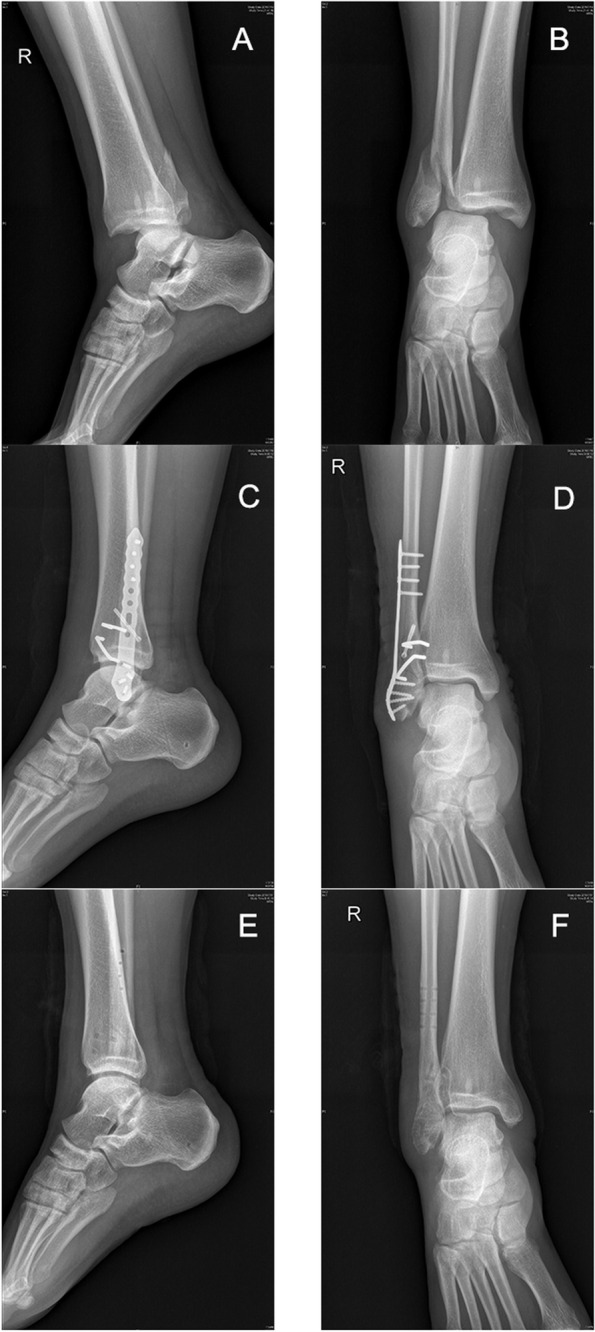
Pre- and postoperative radiographs of a patient in the ASC group. **A**, **B** Fracture of the lateral malleolus and ligament injury of the distal tibiofibular joint were obvious. **C**, **D** After reduction and fixation of the lateral malleolar fracture, ASC was added to reduce and fix the ligament relationship of the distal tibiofibular joint. **E**, **F** The figure shows the patient's fracture reduction and stability more than one year after the removal of all fracture internal fixations

**Fig. 3:**
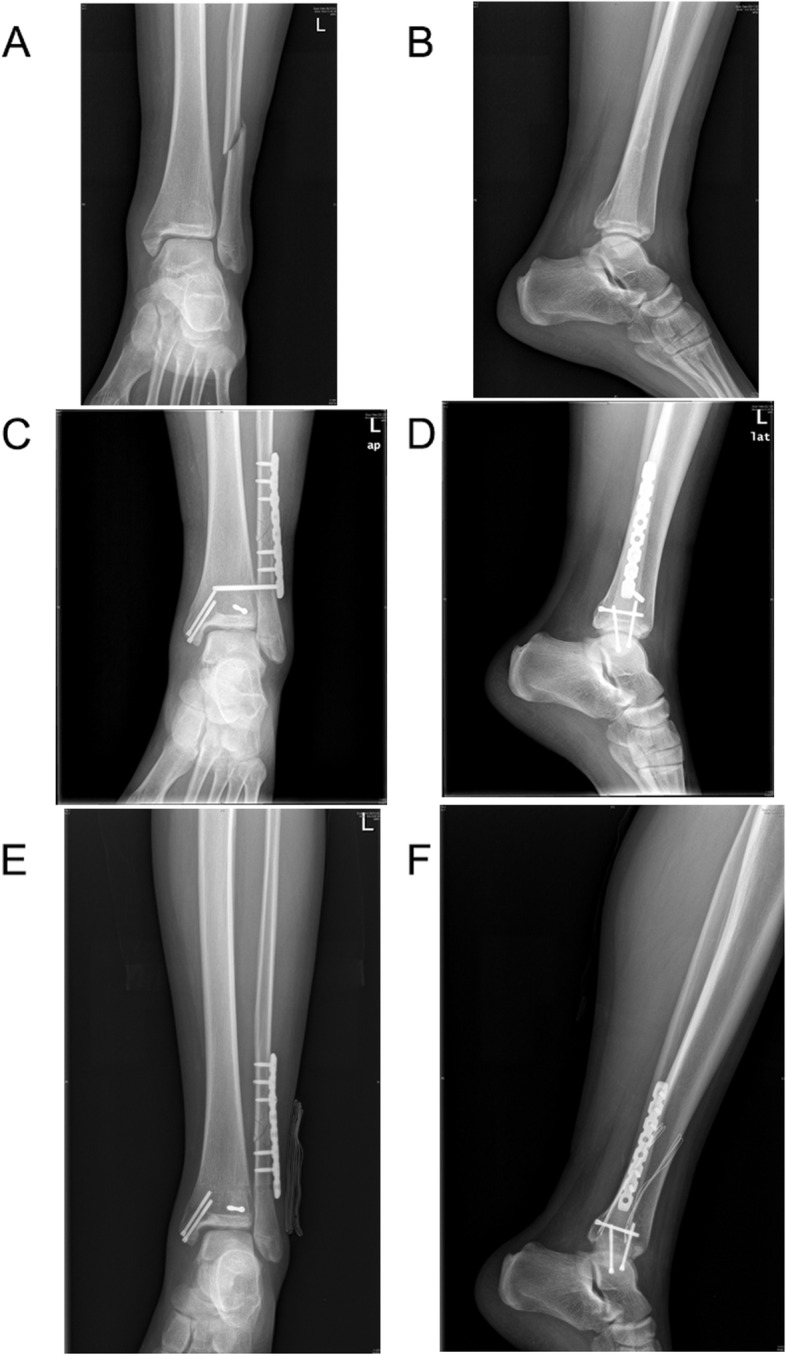
Pre- and postoperative radiographs of a patient in the SCREW group. **A**, **B** Fragments of the internal and external and posterior ankles and ligaments of the distal tibiofibular joint were evident. **C**, **D** After the reduction and fixation of the fracture, a screw was added to reduce and fix the ligament relationship of the distal tibiofibular joint. **E**, **F** The figure shows the patient's fracture reduction and stability approximately one month after the removal of all fracture internal fixations

In the group of patients divided by surgical intervention, we selected two patients, and after signing informed consent, pre- and postoperative X-ray fluoroscopy results were used to show the practical effect and prognosis of the two surgical methods (Fig. 2 for ASC and Fig. 3 for SCREW fixation).

## Discussion

After comparing with the follow-up results of the SCREW group, we found that either the screw or the ASC device used to fix the distal tibiofibular joint can obtain satisfactory prognostic results. A slight difference is that in the statistics of complications, we found that the incidence of symbolic hardware and soft tissue irritation and Malreduction has decreased. Simultaneously, because the ASC device does not require early removal of the fixation device, the need for secondary surgery is reduced in the short term and the chance of loss of reduction is also reduced.

With our understanding of ankle joint fractures combined with distal tibifibular syndesmosis ligament injuries, there is no doubt about the necessity of timely and effective stabilization of the distal tibifibular joint [23]. Solid and stable lag screws can effectively recover from the tibiofibular joint and syndesmosis ligament repair. According to the surgeon's habits, after resetting the tibiofibular joint, 3 or 4 layers of cortical bone can be selected to achieve the maximum fixation effect [24, 25]. However, the problems exposed by this fixed method are similar to a chain reaction. Early patients reported cases of lag screw fracture (3 cases) and loose screw fracture (13 cases) [6]. While people are still studying whether penetrating several layers of the cortex, inserting a few screws, or leaving broken screws in the body affects the prognosis of patients [26, 27], more scholars believe that an intact, solid screw limits the slight displacement between the tibiofibular bones, which is the culprit for many complications of the distal tibiofibular syndesmosis ligament group [28]. Statistics show that the incidence of OA (osteoarthritis) in SCREW group patients (13.8%) is significantly higher than that in ASC group. Screw fixation that penetrates three or even four layers of cortical bone is solid and effective, and it also completely limits the microscopic changes in the joint. Activity. Violation of the normal physiological function of joints may be the main reason for the high incidence of OA. Except for the occurrence of definite diagnosis of osteoarthritis as an evaluation of the treatment effect, the postoperative prognostic scores of the two patients we quoted are also used as important parameters for follow-up prognosis. OMAS itself is a professional score related to the ankle joint, and SF-36 is also a commonly used scoring system for evaluating the patient's prognosis comfort [20–22]. Solid joint fixation often leads to various degrees of complications in the joint. The removal of inferior tibiofibular screws between 1 and 2 months after surgery is recommended by many professors and literatures. A second operation for implant removal could lead to potential infections, an increased cost to the patient, missed work days, or other complications [29, 30]. Moreover, a small prospective trial suggested that the screw fixation method resulted in malreduction and loss of reduction [31]. Beumer et al. showed that syndesmosis screw fixation did not prevent syndesmosis separation under normal weight bearing [32]. Therefore, the separation of syndesmosis is sometimes inevitable after the screw is removed. Traditional X-ray and intraoperative fluoroscopy are not accurate in assessing the reduction of tibiofibular syndesmosis ligament injury, especially in determining whether the fibula is externally rotated.

ASC is widely used in other medical disciplines and provides inspiration [10–17]. It is a solid and reliable rigid fixation screw that can replace a fixed distal tibiofibular joint in new ways. The results of long-term follow-up and early distal tibiofibular joint fixation show that the removal rate of the device is 1.38% (100% for screws). Of the two patients in the group who had the internal fixation device removed early, one patient strongly requested early removal of the internal fixation device because of his own wishes. Another patient developed local hard object irritation, and the implant had to be removed after the patient's reduction and recovery was assessed. As mentioned above, the existence of the broken screw in the body in the report does not affect the prognosis of the patients [8]. However, broken screws cannot be easily removed, which causes inner anxiety. The investigators found that four patients had ruptured the ASC fixator during follow-up, and superficial fixation enabled the operator to completely remove the ASC device without difficulty. In addition, in the postoperative imaging examination and evaluation of the patient, no malreduction was found until the end of the follow-up. Only one patient had osteoarthritis symptoms in the late follow-up.

Currently, a suture button system as a device-fixed distal tibiofibular joint has been in use for several years by fixing the nonrigid limit under significant activity tibiofibular syndesmosis ligaments and repairing tibiofibular syndesmosis ligaments [33].

The application of this system reduces the risk of poor joint reduction [5]. In terms of its later functional score, compared with patients in the screw group, patients in the suture button system group had a higher OMAS score, a lower VAS pain score, and a stronger VAS function [34–37]. Allowing the physiological range of movement of the distal tibifibular joint can theoretically avoid the removal of the internal fixation device and reduce the incidence of adverse events such as damage to the fixation device and loss of reduction after early removal. Even though the TightRope system was initially presented as a device that did not need removal, the rate of implant removal might be as high as 25% [38]. In the current review it was 10% on average. Several authors have already made suggestions to lower the rate of implant irritation and subsequent removal [39–41]. In addition, the main material of the suture button device is an ultrahigh molecular weight polyethylene loop, which can become loose under load, even if the tension has been adjusted during surgery. Forsyth et al. found that in their model of distal tibiofibular syndesmosis ligament injury, no amount of force applied was able to maintain distal tibiofibular syndesmosis ligament reduction by a suture button device. The study found that the suture button device loosened after the patient had been fully weight-bearing for some time [42, 43].

Researchers have not stopped at the problem of avoiding early removal of internal fixation, so absorbable screws have emerged. Polyosteolytic acid degrades more slowly than other polymers and retains strength for longer periods, so it may be more suitable for the fixation of tibiofibular syndesmosis ligament. However, studies have shown that absorbable screws have failed to effectively reduce the incidence of complications compared with traditional screws. Removal rates of internal fixation are higher [44–48]. Moreover, Bostman et al. found that the reaction rate of polyglycolic acid-related foreign bodies reached 5.3% and that of polylactic acid foreign bodies reached 0.2% [49].

ASC fixtures perform well in other medical disciplines, giving us encouragement. We tried to use nitinol alloy memory ASC internal fixation to treat distal tibiofibular syndesmosis ligament injury. Medical nitinol alloy is an alloy material with a special shape memory function at a specific temperature, and its deformation temperature is 0 °C- 5 °C and recovery temperature is 30 °C-40°C. It has the characteristics of high strength, strong bending and torsion resistance, fatigue resistance, corrosion resistance, nontoxicity and good histocompatibility. The restorative force generated by the memory alloy at a body temperature of 37 °C is used to generate dynamic and continuous compressive stress to achieve the fixation effect. Nitinol alloy ASC, as one of its important applications in recent years, has been widely used in the treatment of multiple fractures. Navicular bone, shoulder blades, and collarbone non-load-bearing bone can be used independently to obtain reliable fixation. In the tibia platform, the femoral condyle position can also adapt to changeful fracture lines, such as auxiliary steel, and obtain satisfactory curative effects [10–17]. Nitinol memory ASCs were used for the first time in the treatment of tibiofibular syndesmosis ligament injury in this group. Compared with other internal fixation materials, it has the following characteristics: (1) According to the different types of ankle fractures, the anterior or posterior part of the tibiofibular joint was exposed through the lateral malleolar fracture incision. Imminent reduction under direct vision without additional incisions helps to avoid poor or excessive reduction of the tibiofibular joint (Fig. 1). (2) The ASC design medium, a variety of models, generally medium (20 mm wide) can meet most of the cases, the two claws of the ASC range 12 to 15 mm, can pass through a layer of cortical bone hook into the medullary cavity. The ASC device can limit the gap between the joints by immobilizing the joints with a single layer of cortex, while maintaining slight movement. The incidence of broken is also reduced; (3) The continuous compressive stress of ASC at normal body temperature may be one of the important factors of high healing rate and short healing time in this group of cases. (4) ASC fixation of the tibiofibular joint does not require a second operation to remove the joint in advance. In some cases, fractures occurred after ligament healing, and there was no loss of reduction. The two broken segments were located on both sides of the tibiofibular joint and did not enter the bone marrow cavity. (5) ASC is located on the surface of the anterior distal tibiofibular syndesmosis ligament or the posterior syndesmosis ligament, which is deep on the surface, without complications such as soft tissue irritation or internal fixation discomfort.

In conclusion, in this study, we adopted two surgical methods to draw conclusions after comparing prognosis and complications. We failed to set all the operation methods, and the second follow-up time was not long enough. Although there are many limitations in our research, we can still obtain guidance from them. The results of our series suggest favourable treatment outcomes of syndesmosis injuries with ASC fixation. The ASC group was used to fix the inferior tibiofibular syndesmosis ligament injury. We fixed the joint by only penetrating the single layer of cortical bone, and only one claw was screwed into the tibia and fibula. This fixation method can effectively limit the gap between the tibia and fibula (Although it shrinks after warming, the device is a monocortical fixation, and the spacing between the compressed tibia and fibula depends on the location of the drill hole in the monocortical fixation). The relative mobility of the joints, but the unicortical fixation allows slight movement between the joints, which is also consistent with the physiology of the inferior tibiofibular joint. According to the results of the initial control study, the ASC may not only serve as an effective device providing continuous concentrative compression for syndesmosis lesion healing but also retain the micromovable feature, thereby aiding the restoration of joint function permitting early rehabilitation with a low incidence of postoperative hardware complications such as breakage, irritation and looseness. Furthermore, a large, prospective, randomized controlled trial and bio-mechanical testing are underway for a future study.

## Data Availability

The table in the article has provided the data of this study in more detail, If you need more information, please contact the newsletter and the first author of this article.
